# Management of Typical and Atypical Pulmonary Carcinoids Based on Different Established Guidelines

**DOI:** 10.3390/cancers10120510

**Published:** 2018-12-12

**Authors:** Rohit Gosain, Sarbajit Mukherjee, Sai S. Yendamuri, Renuka Iyer

**Affiliations:** 1 Division of Hematology & Oncology, Roswell Park Comprehensive Cancer Center, University at Buffalo School of Medicine, Buffalo, NY 14203, USA; rohit.gosain@roswellpark.org (R.G.); sarbajit.mukherjee@roswellpark.org (S.M.); 2 Division of Hematology & Oncology, University of Oklahoma Health Sciences Center, Oklahoma City, OK 73104, USA; 3 Depart of Thoracic Surgery Oncology, Roswell Park Comprehensive Cancer Center, University at Buffalo School of Medicine, Buffalo, NY 14203, USA; sai.yendamuri@roswellpark.org

**Keywords:** lung NET, atypical carcinoid, typical carcinoid, pulmonary neuroendocrine tumors, guideline, adjuvant therapy

## Abstract

Neuroendocrine tumors (NETs) are a group of malignancies that originated from neuroendocrine cells, with the most common sites being lungs and the gastrointestinal tract. Lung NETs comprise 25% of all lung malignancies. Small cell lung cancer is the most common form of lung NETs, and other rare forms include well-differentiated typical carcinoids (TCs) and poorly differentiated atypical carcinoids (ACs). Given the paucity of randomized studies, rational treatment is challenging. Therefore, it is recommended that these decisions be made using a multidisciplinary collaborative approach. Surgery remains the mainstay of treatment, when feasible. Following surgery, various guidelines offer different recommendations in the adjuvant setting. In this paper, we describe the adjuvant management of lung NETs, as recommended by different guidelines, and highlight their differences. In addition to that, we also discuss the management of metastatic lung NETS, including the use of peptide receptor radionucleotide therapy.

## 1. Introduction

Neuroendocrine tumors (NETs) consist of a heterogeneous group of malignancies originating from neuroendocrine cells, with most common sites being lung, small intestine, and rectum [1]. In recent years, the incidence of lung NETs has increased in comparison with other NETs [1]. This increase in detection may be attributable to better diagnostic techniques and increased screening.

Lung NETs range in aggressiveness from low-grade typical carcinoid (TC) and intermediate-grade atypical carcinoid (AC) to the high-grade large cell neuroendocrine carcinoma (LCNEC) and small cell lung carcinoma (SCLC) [2,3]. TC have excellent prognosis post-surgery, where five-year overall survival (OS) ranges from 87% to 100% [4,5,6]. AC, on the other hand, has a lower five-year OS in comparison to TC, which mainly ranges from 40% to 90% [5,7,8]. High-grade lung neuroendocrine carcinoma, SCLC, and LCNEC have similar OS trends [9]. Limited-stage SCLC five-year OS is 10–13%, while the extensive stage is 1–2% [10]. Neuroendocrine tumors comprise 25% of all primary lung cancers, while the remaining 75% consists of non-small cell carcinoma (NSCLC) [11]. The most common lung NET is SCLC (20%), followed by LCNEC (3%), and rare incidence of TC (2%), and AC (0.2%) [11].

Lung NETs (TC and AC) are rare diseases. The etiology for this rare type of NET is not well known, and the majority is considered to occur sporadically. Approximately 5% to 10% of cases have been associated with multiple endocrine neoplasia type 1 (MEN1) [12]. There has also been evidence of a rare entity called familial pulmonary carcinoid tumors. Oliveira et al. reported two sets of first-degree relatives diagnosed with primary lung NETs with no clinical or genetic features of MEN1 [13].

There is a paucity of randomized studies, which makes the diagnosis and treatment of lung NETs challenging. The goal of treatment is to essentially control tumor growth and the secretory pattern of NET cells. Given the complexity of the disease, a multidisciplinary approach is used, with surgery being the mainstay of treatment when feasible. There is no consensus for adjuvant therapy in lung NETs. Both retrospective studies and prospective trials in the adjuvant setting are lacking. Here, we provide a comprehensive review of the literature and a comparative summary of current guidelines on the management of lung NETs. 

## 2. Epidemiology

Lung NETs are a rare group of tumors with an estimated age-adjusted incidence of 0.2 to 2 cases per 100,000 population in the United States and Europe [1,14,15]. However, there has been an uptrend in the incidence of lung NETs lately, with an average increase of 6% every year for the last 30 years [5,14]. TCs are more common than ACs, comprising almost 90% of well-differentiated lung NETs [11].

Lung NETs are more prevalent in women, and in terms of race, they are more prevalent in Caucasian, Hispanic, and Asian populations [1,14,16,17,18]. The average age at diagnosis is 40–60 years, with a mean age of diagnosis at 45 years for TC and 55 years for AC [7,19,20,21,22].

## 3. Diagnosis & Classification

Lung NETs consist of a wide variety of neoplasms, ranging from very aggressive ones with a very poor prognosis, to indolent malignancies with long-term life expectancy. The World Health Organization (WHO) classifies lung neuroendocrine pathologies into four variants: TC, AC, LCNEC, and SCLC.

Lung NETs often are asymptomatic or present with generalized symptoms of cough, wheezing, asthma, and chronic obstructive pulmonary disease [2,12]. Depending on the location of the tumor, lung NETs can cause chest pain and hemoptysis as well [15,23,24]. Unlike gastroenteropancreatic NETs, lung NETs present with carcinoid syndrome in fewer patients: usually less than 10% of all patients [25]. In rare scenarios, Cushing syndrome and acromegaly from the overproduction of adrenocorticotropic hormone and growth hormone-releasing hormone respectively, are observed as well [26,27,28,29]. Lung NETs are usually diagnosed at an earlier stage. However, in advanced stages, TC usually metastasizes to liver and bone, while AC metastasizes to liver, bone, brain, soft tissue, and adrenal/spleen [30].

The two important criteria that help classify lung NETs are: the number of mitoses per 2 mm^2^ of a viable area around the tumor, and the presence or absence of necrosis [31,32]. TCs are tumors with less than two mitoses per 2 mm^2^ of a viable area of the tumor, with the absence of necrosis, while ACs have two to 10 mitoses per 2 mm^2^ with a presence of focal necrosis [31,32]. SCLC and LCNEC are both high-grade, and have more than 10 mitoses per 2 mm^2^, as well as an extensive degree of necrosis [31,33].

Ki-67 antigen immunohistochemistry (IHC), which defines the cell proliferation labeling index, helps in differentiating low-grade lung NETs (<20%) from high-grade NETs (≥20%) in small crushed biopsies [31,34]. Generally, the Ki-67 proliferation rate of TC is less than 2%, while the AC is less than 20%. Given such a distribution overlap of Ki-67 expression between TCs and ACs, the Ki-67 index does not further differentiate between well-differentiated pulmonary carcinoids [15,35,36]. Walts et al. highlighted the Ki-67 indices as being significantly different in TC and AC, but having considerable overlap. They also noticed that the addition of the Ki-67 index to histological diagnosis does not provide significant prognostic value [36]. In addition to that, consensus for assessing the Ki-67 index using an appropriate technique is also lacking, as there exists about four methods: digital image analysis, manual counting, eyeball estimation, and number of cells [15]. While Ki-67 staining is required in gastrointestinal NET classification and does not overlap between grades and aids in prognosis, it is not mandated to report Ki-67 in lung NETs.

The WHO classification recommends checking for chromogranin A, synaptophysin, and CD56 markers to confirm lung NET diagnosis [5,31]. Although TTF-1 is used, it is often negative in typical and atypical carcinoids. In addition to these markers, imaging techniques also play a major role in identifying the tumor extent, and also help in staging. Computed tomography (CT) aids in localization and staging of the tumor, while MRI (magnetic resonance imaging) helps detect bone or liver metastases [15]. Imaging techniques with radiolabeled somatostatin analogs (SSA) help, as most patients with TCs and ACs express somatostatin receptors [37]. With the use of positron emission tomography (PET) imaging, PET tracers that are labeled somatostatin analogues have been developed [38]. This recent development has sparked interest in a new imaging modality, Gallium 68-1,4,7,10-tetraazacyclododecane-1,4,7,10-tetraacetic acid-octreotate positron emission tomography/computed tomography (^68^Ga-DOTATATE PET/CT), in the management of NETs [38]. ^68^Ga-DOTATATE PET/CT has been demonstrated to be a great tool. One study found that the affinity of DOTATATE in binding to somatostatin receptors is 10-fold higher than that of octreotide [39]. Several studies have looked at the sensitivity and specificity of ^68^Ga-DOTATATE PET/CT, and have shown promising results. Comparing ^68^Ga-DOTATATE PET/CT to gold standard pathology, ^68^Ga-DOTATATE PET/CT identified 29 of 36 patients (sensitivity, 81%) with NET, and excluded the presence of NET in 61 of 68 non-NET patients (specificity, 90%) [40]. Another study showed that ^68^Ga-DOTATATE PET/CT identified NET recurrence in 26 of 29 patients (sensitivity, 90%) and excluded NET in 28 of 34 patients (specificity, 82%) [41]. When comparing ^68^Ga-DOTATATE to Octreoscan, the ^68^Ga-DOTATATE scan identified significantly more lesions in comparison to Octreoscan (*p* < 0.001) [42]. ^68^Ga-DOTATATE was further tested in neuroendocrine patients who had negative or equivocal findings on Octreoscan, and ^68^Ga-DOTATATE was found to be positive in 41 of 47 patients [42].

In this regard, PET/CT using ^68^Ga-DOTATATE is preferable to Octreoscan, if available, as the former has better resolution, decreased scanning time, higher binding affinity to somatostatin receptors, and high sensitivity and specificity [43,44,45].

## 4. Treatment

### 4.1. Localized

Surgical resection is the treatment of choice for localized pulmonary carcinoids. All of the guidelines endorse surgical resection in localized TCs and ACs, as it has been shown to yield five-year survival rates of 90% in TCs and 70% in ACs [8,15,24,46,47,48,49]. This evidence has been mainly derived from retrospective analyses, given the lack of prospective studies. The European Society of Thoracic Surgeons Neuroendocrine Tumours Working Group showed that patients who underwent resection for TC were associated with a five-year survival rate of 94% [50]. On the other hand, a retrospective database analysis of 441 AC patients showed that surgical resection leads to a three-year survival of 67% [51].

The goal of surgery is to conserve as much of the normal lung tissue as possible while performing the resection with a tumor-free resection margin (R0), which is associated with good prognosis [52]. Given the advances in imaging modalities, most patients are detected in earlier stages. Therefore, local resection is possible in most cases. In peripherally localized tumors, wedge resection is favored, and is often sufficient [53]. However, if the tumor is localized in the central airway, one has to undergo complex resections with angioplasty/bronchoplasty [54]. Fox et al., in their SEER database review, highlighted the common surgical approaches in pulmonary NETs. Lobectomy (1669; 51.2%) was the most common surgical approach, while another major approach was sublobar resection with wedge resection or segmentectomy (784; 24.1%) followed by ablation, pneumonectomy, bronchoplasty, or extended resection [55]. Compared to the lobectomy subgroup, sublobar resection patients comprised older patients (>60 years), and had lower tumor (T)-stage and nodal (N)-stage [55]. Patients who underwent sublobar resection had statistically noninferior overall survival compared the to lobectomy subgroup at two years and five years [55]. Yendamuri et al. also confirmed these findings, where they showed that lobectomy for the TC tumors is not superior to sublobar resection, as long as patients get complete resection, and adequate mediastinal staging is performed [56]. Given the involvement of adjacent lymph nodes in locally advanced pulmonary NETs, current guidelines recommend either sampling or lymph node dissection during the local resection (Table 1).

Currently, there is no consensus on adjuvant therapy in pulmonary carcinoids as per the North American Neuroendocrine Tumor Society (NANETS), National Comprehensive Cancer Network (NCCN), European Neuroendocrine Tumor Society (ENETS), or European Society of Medical Oncology (ESMO) guidelines [24,46,47]. The NCCN guidelines recommend considering adjuvant cisplatin and etoposide with or without radiation in stage III ACs [47]. On the other hand, ENETS recommends considering adjuvant treatment in ACs with positive lymph nodes, and no adjuvant therapy for TCs [57]. Table 1 below highlights how different guidelines vary in pulmonary carcinoid management.

These differences between the guidelines with respect to adjuvant therapy recommendations mainly stem from the lack of prospective data. Retrospective analyses have shown no overall survival benefit with adjuvant therapy. Nussbaum et al. conducted a retrospective analysis, looking at the National Cancer Database (NCDB) from 1998 through 2006 for TC patients with lymph node involvement who underwent surgery followed by adjuvant chemotherapy versus observation [58]. After propensity matching, there was a trend toward inferior five-year survival for patients who received adjuvant chemotherapy versus observation (69.7% versus 80.9%; *p* = 0.096) [58].

Similarly, in node-positive AC patients, there was no survival advantage in patients who underwent surgery followed by adjuvant chemotherapy versus observation (47.9% versus 67.1%; *p* = 0.46) [59]. Westin et al. reported similar findings in their NCDB analysis from 2004–2012, where they found no survival benefit of adjuvant chemotherapy in TC and AC patients; however, adjuvant therapy was beneficial in large cell neuroendocrine tumors [60]. Prospective clinical trials are needed to confirm these findings.

### 4.2. Locally Advanced

There is limited data on combination therapy for stage IIIA and IIIB pulmonary NETs. As a result, a multidisciplinary approach is recommended. The NCCN guidelines highlight that if surgical resection is not feasible or following margin-positive resection for a stage IIIA and IIIB TC, radiation therapy with or without platinum-based chemotherapy is considered [47]. For AC in similar settings, concurrent radiation therapy with or without concurrent chemotherapy with platinum and etoposide is recommended [47]. The use of chemoradiation is considered to be more beneficial in aggressive tumors and high mitotic burden, which mainly stems from retrospective studies [30,61]. On the other hand, the ENETS guidelines indicate considering adjuvant therapy in AC patients with positive lymph nodes, especially in cases of high proliferative index [57]. Given excellent prognosis post-surgery, the use of adjuvant therapy in TC patients is not recommended by the ENETS guidelines [57].

### 4.3. Metastatic

The treatment strategy for advanced stage pulmonary carcinoids is not curative, and is directed at controlling symptoms from the tumor burden or hormonal production and slowing tumor growth. Dedicated trials for pulmonary NETS have been initiated only recently. Therefore, most treatment recommendations come from case studies, case series, or trials focused on well-differentiated NETs of other origins, which included lung NETS [15].

### 4.4. Somatostatin Analogs (SSA)

In asymptomatic patients with low tumor burden, watchful waiting may be considered as per the NCCN and ENETS guidelines [15,47]. Alternatively, somatostatin analogs (SSAs) may be used. These agents promote stabilization in up to 30–70% of patients with well-differentiated NETs, as demonstrated in studies that included lung NET patients [62,63,64]. A retrospective study evaluated the role of somatostatin analogs in progressive, metastatic pulmonary NETs [65]. Among the 61 patients included in the study, the best overall response, which was stable disease, was observed in 47 (77%) patients, with an overall median progression-free survival (PFS) and OS of 17.4 (95% CI: 8.7–26) and 58.4 months (95% CI: 44.2–102.7) respectively, while slowly progressive disease and functioning tumors were associated with longer PFS [65]. Similar findings were observed in another single institution study, which evaluated the role of octreotide or lanreotide, a somatostatin analogue, in a total of 35 patients, with five being pulmonary NET [63]. One out of the 35 patients (3%) who were enrolled achieved a partial response, while the disease was stabilized in 20 patients (57%) for a median duration of 11 months (six to 48 months) [63].

The NCCN guidelines on pulmonary NETs recommend the use of octreotide or lanreotide for patients exhibiting symptoms of carcinoid syndrome, or those who have octreotide-positive scans [47]. ENETS guidelines also agree on using SSAs in symptomatic carcinoid patients, in addition to patients with positive somatostatin receptor positive status on Octreoscan and a low Ki-67 proliferative index of <10% [57]. The ESMO and NANETS also support the use of SSAs in symptomatic patients [24,46].

The PROMID study included treatment-naïve well-differentiated metastatic NETs of gastrointestinal (GI) or unknown primary origin patients. Patients who received somatostatin analog, interferon alpha, chemotherapy, or chemoembolization were excluded. Patients were randomized to treatment with 30 mg of octreotide long-acting release (LAR) versus placebo. Patients in the octreotide group were found to have increased progression-free survival (PFS) compared to placebo: 14 months versus six months, respectively (hazard ratio (HR) = 0.34; 95% CI, 0.20 to 0.59; *p* = 0.000072) [66]. Another SSA that showed improvement in PFS compared to placebo is lanreotide. Lanreotide was studied in a phase III randomized placebo-controlled trial (CLARINET), where 204 patients with advanced, well-differentiated, or moderately differentiated enteropancreatic NETs were compared to placebo. In comparison to the PROMID study, the CLARINET study included patients with documented progressive disease. PFS at 24 months was significantly improved in the lanreotide group; 65% versus 33% in the placebo group (*p* < 0.001) [67]. The ongoing SPINET study will evaluate whether the benefit seen in the PROMID and CLARINET studies is also noted in TC and AC patients who have somatostatin receptor positive disease [68]. The only prospective evidence for the use of somatostatin analogue in lung NETs is from the octreotide alone arm of the RADIANT 2 trial. This phase III study randomized 429 patients with hormonally active carcinoid tumors, including lung NETs, to treatment with placebo plus octreotide or everolimus plus octreotide [69]. An exploratory subgroup analysis showed that the median PFS was 5.6 months in advanced lung NET patients who received octreotide alone [69]. However, the inclusion criteria for this study included disease progression within the past year. This reflects a different population compared to other SSA trials, where newly diagnosed patients with possibly more indolent biology were enrolled.

### 4.5. Targeted Therapy

Everolimus, an mammalian target of rapamycin (mTOR) inhibitor, is approved for patients with progressive, well-differentiated, nonfunctional neuroendocrine tumors (NET) of gastrointestinal (GI) or lung origin that are unresectable, locally advanced, or metastatic. The efficacy of everolimus was demonstrated in RADIANT-2 and RADIANT-4 trials. It has been well tolerated in patients when added to octreotide long-acting release (LAR) [70]. The phase III RADIANT 2 study, which included 44 bronchial NET patients, showed a median PFS of 13.6 months in the everolimus and octreotide arm, in comparison to 5.6 months in the octreotide and placebo arm [69]. In the RADIANT-4 study, 302 patients with progressive non-functional gastrointestinal or pulmonary NETs, who progressed within the last six months, were randomized to either everolimus or placebo [71]. The subgroup analysis of 90 lung NET patients [69] demonstrated a median PFS of 9.2 months in the everolimus arm versus 3.9 months in the placebo arm (hazard ratio (HR) 0.48 (95% CI 0.35–0.67), *p* < 0.00001) [71].

### 4.6. Cytotoxic Chemotherapy

There is limited data demonstrating the efficacy of chemotherapy in advanced pulmonary neuroendocrine tumors, and most of the data is derived from small prospective studies or retrospective analysis [30,46,72,73]. Cytotoxic chemotherapy with etoposide, platinum, or temozolomide-based regimens have been tried with moderate success [30]. Cisplatin and etoposide cytotoxic combination chemotherapy has been derived from its use in SCLC (small-cell lung cancer) [47,49]. This cytotoxic combination of cisplatin with etoposide was further looked at in three small retrospective studies, where metastatic pulmonary NET patients demonstrated an overall response rate of 20–25% [61,74,75]. Additionally, Fjallskog et al. showed cisplatin and etoposide to be a successful combination for foregut carcinoid (lung and thymus) patients in a prospective study [76]. Cisplatin and etoposide were administered in 18 patients with foregut carcinoid patients who progressed on first or second-line treatment [76]. The study showed that two of the five patients with AC (40%) and five of the 13 patients with TC (39%) had a radiographic response, while the median response duration was nine months, with a range of six to 30 months [76].

An oral alkylating agent, temozolomide, has been studied as a single agent or in combination with other agents. A retrospective study involving 13 bronchial carcinoid patients, showed partial radiological response in four patients (31%), while four patients had stable disease (31%) [77]. Another retrospective study by Crona et al. confirmed these findings where of the 31 patients enrolled, 22 patients were evaluated by response evaluation criteria in solid tumors (RECIST) 1.1 [72]. From the selected cohort, three patients (14%) were found to have a partial response, while 11 (52%) patients had stable disease, and progressive disease was observed in seven patients (33%) [72]. The subgroup analyses revealed that TC patients time-to-progression was 11.5 months, with OS of 37.4 months, and for AC patients, time-to-progression was 7.5 months, with an OS of 33 months [72]. Temozolomide in combination with capecitabine has been evaluated in metastatic carcinoid and pancreatic NETs with promising results, where 61% patients had an overall response, with a median PFS of 14 months and an OS of 83 months [78]. Other combinations with temozolomide in carcinoid patients have been tried; one was with thalidomide, and showed a 7% response rate. On the other hand, when temozolomide was combined with bevacizumab, there was no response, and the combination therapy yielded no benefit [79,80].

Given the evidence of response, but not a sustainable one, there is no standard of care therapy in this setting. The NCCN guidelines suggest considering cytotoxic chemotherapy in patients when no other treatment modality is available [47]. The cisplatin–etoposide combination is the preferred systemic therapy in advanced TCs and ACs. The ENETS guidelines support the use of systemic chemotherapy in pulmonary NETs if Ki-67 >15%, rapidly progressing disease, and when the somatostatin receptor imaging is negative [57].

## 5. Peptide Receptor Radionucleotide Therapy (PRRT)

PRRT is an innovative treatment modality to treat inoperable or metastasized, well-differentiated, or moderately differentiated NETs. PRRT therapy with lutetium 177 (^177^Lu)-Dotatate has been used in metastatic NETs, primarily in midgut neuroendocrine tumors [81,82,83,84]. Recently published results of NETTER-1, a phase III randomized trial of 177Lu-DOTATATE versus high-dose octreotide long-acting release (LAR) in inoperable, progressive, metastatic midgut well-differentiated carcinoid tumors, showed that ^177^Lu-octreotate markedly improves PFS (at 20-month intervals, 65.2% in the PRRT group, while 10.8% in the control group), with pending OS results [84]. Additionally, the overall number of deaths was less in the PRRT group in comparison to the control group: 14 versus 26, respectively [84].

There has been recent evidence suggesting the successful use of PRRT in pulmonary NETs, as these tumors express the somatostatin receptor [85]. In a retrospective study, the efficacy of PRRT was evaluated in 19 patients with pulmonary carcinoids. Based on predefined response evaluation criteria, patients were classified as responders or non-responders. In that study, 12 patients (63%) were labeled as responders, with a median OS of 40 months, and seven patients were classified as non-responders [86]. Additionally, two European studies have also demonstrated positive data with the use of PRRT in advanced stage lung NET patients. One Italian retrospective study evaluated 114 patients with advanced stage bronchopulmonary carcinoid patients who got PRRT [87]. Patients were found to have a median OS of 58.8 months and a median PFS of 28 months, while morphological responses (partial response and minor response) were observed in 26.5% of patients, attributing to an increase in OS and PFS [87]. Another European study, involving Dutch patients with gastroenteropancreatic (GEP) and bronchial NETs, evaluated 443 patients treated with PRRT. They observed that the total group’s objective response rate was 39%, while PFS and OS were 29 months (95% CI, 26–33 months) and 63 months (95% CI, 55–72 months), respectively [88]. When the efficacy of PRRT was compared amongst different NETs, the group demonstrated pancreatic NET to be the most responsive, followed by midgut and then lung NET, with median OS of 71 months, 60 months, and 52 months, respectively [88]. On the other hand, Sharma et al. analyzed data from 135 patients who were treated with PRRT, and found the median OS to be 40 months from the first PRRT treatment [89]. Comparing OS since the first PRRT treatment, the investigators found patients with small-bowel NETs to have the maximum OS benefit of 95.4 months, followed by pancreatic NETs, with a median OS of 37.3 months, and lung NETs, with a median OS of 32.4 months [89]. Further prospective studies are needed in order to evaluate the efficacy of PRRT, which seems to be a very promising treatment option in inoperable somatostatin receptor positive pulmonary NETs in terms of symptom control, OR, PFS, OS, and quality-of-life improvement [90,91].

Having such remarkable results with PRRT, the United States (US) Food and Drug Administration (FDA) approved PRRT for the treatment of somatostatin receptor-positive foregut, midgut, and hindgut NET on 26 January 2018. The NCCN guidelines added PRRT as a potential option for metastatic low/intermediate grade GEP NETs, including foregut, midgut and hindgut patients [47]. ENETS guidelines also recommend considering PRRT therapy as an option for patients whose tumors have high uptake on somatostatin receptor scintigraphy [57].

## 6. Future Advancements

In this era of personalized cancer development, in order to develop novel therapies and test various combinations in NETs, it is important to understand the biology of these tumors closely. Studies have demonstrated that NETs are highly vascularized cancers where well-differentiated tumors tend to have increased microvessel density in comparison to poorly differentiated tumors [92,93,94,95]. As a result, multi-tyrosine kinase inhibitors (TKI) such as sunitinib demonstrated increased PFS of 11.4 months in pancreatic NET patients [96]. There has been evidence of positive c-MET staining in ileal as well as non-ileal NETs, making it another potential target for therapy [97]. Knowing such potential targets, a phase II trial tested the use of cabozantinib—a multi-tyrosine kinase inhibitor—and showed promising results in carcinoid tumors and pancreatic NETs, with a median PFS of 21.8 months (95% CI, 8.5–23.0) in the pancreatic NETs group and 31.4 months (95% CI, 8.5-NR) in the carcinoid tumor cohort [98]. Another TKI therapy investigated by Iyer et al. in a phase II study was nintedanib, involving 30 patients with advanced stage metastatic carcinoids on a stable dose of somatostatin analogue. Based on Nintedanib’s activity to inhibit angiogenesis and block the fibroblast growth factor receptor (FGFR) pathway, this oral drug demonstrated PFS and OS of 11 months and 27.6 months, respectively [99]. Other TKI therapies, which are currently under investigation in treating NETs are ibrutinib, sulfatinib, pazopanib, and regorafenib.

Immunotherapy has revolutionized the field of oncology. We have seen remarkable results with immune checkpoint inhibitors (ICIs) in high-grade pulmonary NET/SCLC. Checkmate 032 compared the use of nivolumab 3 mg/kg, nivolumab 1 mg/kg plus ipilimumab 3 mg/kg and nivolumab 3 mg/kg plus ipilimumab 1 mg/kg in 216 SCLC patients who progressed on prior platinum-based regimens. The median overall survival that was observed was 4.4 months (95% CI 3.0–9.3) in the nivolumab arm, while 7.7 months (3.6–18) in the nivolumab 1 mg/kg plus ipilimumab 3 mg/kg arm, and six months (3.6–11) in the nivolumab 3 mg/kg plus ipilimumab 1 mg/kg arm [100]. The activity of the ICIs is being investigated now in low-grade pulmonary NETs and other non-pulmonary NETs. There are active clinical trials to address the role of ICIs in NETs. Some of the ongoing trials are pembrolizumab as a monotherapy in metastatic high-grade NETs; pembrolizumab in combination with lanreotide for GEP-NETs; spartalizumab in well-differentiated pancreatic, gastrointestinal or thoracic origin; a combination of durvalumab plus tremelimumab for advanced gastrointestinal, pancreatic and lung NETs.

## 7. Conclusions

Given the rarity of pulmonary NETS and conflicting evidence in regards to the management, specifically in the adjuvant setting, there is no consensus amongst different guidelines.

In locally advanced pulmonary NET patients, treatment options are confined to surgery and somatostatin analogs (octreotide and lanreotide); however, recent trials have shown benefit with everolimus. The combination of octreotide and everolimus has shown improvement in PFS in pulmonary NET patients. Along with that, we have PRRT as a potential option, which has benefited gastroenteropancreatic (GEP) and lung NET patients. Given the advancements with novel therapies such as PRRT, it is time to test if this therapy can be combined with other established therapies in the field of NETs. There are ongoing trials where PRRT is being combined with capecitabine for GEP NET patients, and another trial where PRRT is being combined with capecitabine and temozolomide in midgut and pancreatic NET patients [101].

Given these current advancements in the management of NETs therapy and promising evidence with new modalities such as immunotherapy, PRRT, and combinatorial regimens, we are making strides in the field with a hope for an unprecedented outcome in NET patients.

## Figures and Tables

**Table 1:** Comprehensive review of the management of localized pulmonary neuroendocrine tumor, mainly atypical carcinoids (AC) and typical carcinoids (TC) by different guidelines. ENETS: European Neuroendocrine Tumor Society, EMSO: European Society of Medical Oncology, NANETS: North American Neuroendocrine Tumor Society, NCCN: National Comprehensive Cancer Network.

Guidelines	NCCN	NANETS	ENETS	ESMO
Surgical Approach	Lobectomy along with mediastinal node dissection or sampling	Bronchial NETS—Sleeve resection, along with lymph node sampling in well-differentiatedd bronchial NETs Thymic NETS—median sternotomy approach with complete tumor resection and mediastinal lymphadenectomy	Lobectomy/Segmentectomy along with a minimum of 6 lymph nodes sampling, min of three to be mediastinal including subcarinal Central airway tumor—lung parenchymal sparing surgery Bronchial sleeve resection or sleeve lobectomy preferred over pneumonectomy Systemic nodal dissection	Inoperable—Bronchoscopic laser excision of intraluminal typical bronchial NETs Lobectomy/sleeve resection, along with systemic nodal dissection
Adjuvant Regimen	Stage IIIA/B ACs radiation +/− chemotherapy	No recommendations	Consider adjuvant therapy in ACs with positive lymph nodes	No recommendations
